# Dose–Response Relationships between Second-Hand Smoke Exposure and Depressive Symptoms among Adolescents in Guangzhou, China

**DOI:** 10.3390/ijerph15050985

**Published:** 2018-05-14

**Authors:** Jingya Huang, Bin Xu, Dan Guo, Ting Jiang, Wei Huang, Guocong Liu, Xiaohua Ye

**Affiliations:** 1 School of Public Health, Guangdong Pharmaceutical University, 283# Jianghai Dadao, Haizhu District, Guangzhou 510310, China; jingyi4597@hotmail.com (J.H.); guodan446@163.com (D.G.); 2 Health Education Section, Guangzhou Yuexiu Center for Disease Control and Prevention, 23# Jiaochang West Road, Yuexiu District, Guangzhou 510055, China; xubin6710@163.com (B.X.); jiangting04@126.com (T.J.); yxqhwei@163.com (W.H.)

**Keywords:** second-hand smoke, smoking, depressive symptoms, adolescents

## Abstract

There has been little focus on the possible association between second-hand smoke (SHS) exposure and depressive symptoms among adolescents. Thus, this study aimed to explore the dose–response relationships between SHS exposure and depressive symptoms among adolescents and differentiate these associations in setting-specific exposure and severity-specific outcomes. A cross-sectional study was conducted using a stratified cluster sampling method to obtain a representative sample of high school students in Guangzhou, China. Depressive symptoms were measured using the Center for Epidemiologic Studies Depression Scale. Univariable and multivariable logistic regression models were used to explore the potential associations between SHS exposure and depressive symptoms. Among 3575 nonsmoking students, 29.6% were classified as having probable depressive symptoms and 9.6% had severe depressive symptoms. There were monotonically increasing dose–response relationships between setting-specific (public places, homes, or indoor/outdoor campuses) SHS exposure and severity-specific (probable or severe) depressive symptoms. When examining these relations by source of exposure, we also observed similar dose–response relationships for SHS exposure in campuses from smoking teachers and from smoking classmates. Our findings suggest that regular SHS exposure is associated with a significant, dose-dependent increase in risk of depressive symptoms among adolescents, and highlight the need for smoke-free environments to protect the health of adolescents.

## 1. Introduction

The Centers for Disease Control and Prevention reported that there is no risk-free level of second-hand smoke (SHS) exposure [1]. The latest retrospective analysis of the worldwide burden of disease attributable to SHS exposure indicated that 40% of nonsmoking children are exposed to SHS [2]. In China, 740 million (including 180 million children) nonsmokers are exposed to SHS [3]. Global youth tobacco surveillance also reported that nearly half of adolescents are exposed to SHS in homes (42.5%) or in public places (55.1%) [4].

There is increasing evidence suggesting that smoking may cause depressive symptoms or depression [5,6]. It is conceivable that nonsmokers exposed to a high level of SHS may also experience depression as a result. Additionally, growing evidence suggests that SHS may be associated with depression through its effects on chronic diseases, chronic stress, and the dopamine system [7,8,9,10,11,12,13]. Based on the above findings, we hypothesized that there may be a potential relationship between SHS exposure and depressive symptoms. Epidemiology evidence about the relation between SHS exposure and depressive symptoms has been reported mainly among adults [14,15,16,17,18,19], and current evidence is inconsistent. A few studies have demonstrated significant relations [14,15,16,17,18], while a nonsignificant relation was found in another report [19]. Compared to the number of studies on adults, few studies report on this relation in adolescents [20,21]. Furthermore, there is still no evidence to show the effects of campus SHS exposure on depressive symptoms among adolescents. Therefore, the present study aimed to explore the potential dose–response relationship between SHS exposure and depressive symptoms among adolescents and differentiate this relationship in setting-specific exposure and severity-specific outcomes to make exposure and outcomes clearer.

## 2. Methods

### 2.1. Ethics Statement

The study was approved by the Ethics Committee of Guangdong Pharmaceutical University, and it was performed in accordance with the approved guidelines (2016–2017). This survey was qualified as involving no risks to study participants. The goals of the study were given to the participants and they expressed their willingness to participate. Written informed consents were obtained from parents or guardians of participants.

### 2.2. Study Design and Sampling

The target population was high school students. A cross-sectional study was conducted in Guangzhou, China between March and April 2016. Note that high schools in most parts of China are generally rated as key schools (or prestigious schools) and ordinary schools (or nonprestigious schools) according to education level and teaching quality. Therefore, this study used a stratified cluster sampling process to obtain a representative sample. First, all high schools were divided into two categories (prestigious or nonprestigious schools). Three high schools were randomly sampled from prestigious schools and four high schools were randomly sampled from nonprestigious schools, which were selected with probability proportional to the number of the schools. Second, classes in the selected schools were randomly sampled proportional to the school enrollment size, and all students in the sampled classes were eligible to participate.

After obtaining informed consent, eligible students were asked to complete a face-to-face survey conducted by trained interviewers. A total of 3833 participants were interviewed, of whom 3657 (95.4%) were willing to participate in this survey. The remaining 176 (4.6%) participants refused to participate since they had to take part in extracurricular training during the investigation. Among 3657 study participants, 3575 (97.8%, 3575/3657) participants were nonsmokers and 82 (2.2%, 82/3657) were smokers.

### 2.3. Data Collection and Quality Control

All interviewers in each school were trained to ensure that the survey was carried out according to the protocol and that operation procedures were identical across all schools. In order to evaluate the feasibility of this investigation, a pilot study was carried out before formal investigation. Two levels of a quality control system (including quality controller and quality leader) were used to check for potential errors in the questionnaires. Data quality was also assured by using double entry procedures to automatically detect data entry errors. Before carrying out statistical analyses, raw data was cleaned to detect and correct (or remove) corrupt or inaccurate records.

### 2.4. Study Variables

**Depressive symptoms**—The outcome variable was self-reported depressive symptoms measured by the Chinese version of the Center for Epidemiologic Studies Depression (CES-D) Scale [22], which is widely used in healthy adults and adolescents [23,24,25]. The 20-item CES-D Scale measures the levels of depressive symptoms experienced in the past week. The scores of CES-D range from 0 to 60, and the cutoffs of 16 and 25 represent the thresholds for probable depressive symptoms (CES-D ≥ 16) and severe depressive symptoms (CES-D ≥ 25), respectively [26]. Cronbach’s alpha for the CES-D scale in this study was 0.854, suggesting good internal consistency of the questionnaire.

**SHS exposure**—The main independent variable was self-reported SHS exposure, which was defined as nonsmokers’ inhalation of the smoke exhaled from smokers on at least one day a week in the past 7 days [27]. Participants were asked if they had smoked over 100 cigarettes in their lifetime [28,29,30], and those responding “no” were defined as nonsmokers. The nonsmokers were asked if they had SHS exposure in public places, homes, indoor campuses, and outdoor campuses, respectively. Frequency of SHS exposure was reported as a continuous variable (days/week), and was also categorized into three groups: <1 day/week (no exposure), 1–4 days/week, and 5–7 days/week.

**Covariate variables**—Covariates including potential mediators and confounders were chosen a priori on the basis of a literature review. Covariates in this study included gender, grade (4–5 or 1–2), only child (yes or no), monthly pocket money (<¥100, ¥100–399, or ≥¥400), prestigious schools (yes or no), parents’ education (neither from high school, one from high school, or both from high school), negative life events (yes or no), and disease history (0, 1, 2, or ≥3). Negative life events were measured by a response of ‘yes’ to any of the following events occurring in participants’ families in the past month: death of family members, violent/suicidal/criminal behaviors of family members, separation from parents, severe medical problems, accident/disaster, theft, financial problems, or poor housing. For the question of disease history in the past month, participants were asked if they had the following diseases or symptoms: asthma, shortness of breath, frequent coughing, feeling of discomfort in the throat, irritation in eyes, irritation in nose, or other diseases.

### 2.5. Statistical Analysis

All data were entered in duplicate into the EpiData version 3.1 database (The EpiData Association, Odense, Denmark), and then every inconsistency was checked by the consistency test. Univariable and multivariable logistic regression models were used to calculate the odds ratios (ORs) and 95% confidence interval (CIs) for evaluating the potential relationship between SHS exposure and depressive symptoms. Potential confounders were controlled by a review of putative risk factors for depressive symptoms and a 10% or greater change in the β coefficients for SHS exposure between the crude and the adjusted models. Linear trends of SHS exposure were assessed by modeling exposures as continuous variables (arithmetic or logarithmic scale) or ordinal variables in the logistic models. We defined a two-sided *p*-value of <0.05 as being of statistical significance. All statistical analyses were performed using Stata version 14.0 (StataCorp LP, College Station, TX, USA). For this study, only nonsmokers were included in the analyses.

## 3. Results

### 3.1. Characteristics of the Sample

Among 3575 nonsmokers, the mean age was 15.0 ± 1.8 years, and 49.1% were female students. As to depressive symptoms, 1058 (29.6%) were classified as having probable depressive symptoms and 343 (9.6%) were classified as severe depressive symptoms. SHS exposure was highest in public places (49.5%), followed by exposure in homes (34.5%), in outdoor campuses (29.2%), and in indoor campuses (22.7%) (Table 1).

### 3.2. Relation between Binary SHS Exposure and Probable Depressive Symptoms

In contrast to no exposure (Table 2), students with indoor SHS exposure experienced a significant prevalence of probable depressive symptoms (OR = 1.38; 95% CI 1.16–1.63, for SHS in indoor public places; OR = 1.24; 95% CI 1.04–1.47, for SHS in homes; OR = 1.57; 95% CI 1.29–1.92, for SHS in indoor campuses). When examining the relations by source of exposure, there were similar positive relations for SHS exposure in indoor campuses from smoking teachers (OR = 1.43; 95% CI 1.15–1.78) and from smoking classmates (OR = 2.12; 95% CI 1.64–2.74). We note that the effects of SHS exposure in outdoor campuses cannot be ignored. There was a significant association between SHS exposure in outdoor campuses and probable depressive symptoms (OR = 1.49; 95% CI 1.24–1.79), and similar positive associations were observed for SHS exposure in outdoor campuses from smoking teachers (OR = 1.65; 95% CI 1.34–2.04) and from smoking classmates (OR = 1.55; 95% CI 1.26–1.91).

### 3.3. Relation between Frequency of SHS Exposure and Probable Depressive Symptoms

Table 3 reveals the frequency–risk relationships between SHS exposure and probable symptoms. Ordinal frequency of SHS exposure was positively associated with prevalence of probable depressive symptoms in a dose–response manner (*p* for linear trend <0.001, for SHS in indoor public places, SHS in homes, SHS in indoor campuses, or SHS in outdoor campuses). Similarly, there were monotonically increasing frequency–risk relationships between continuous frequency of SHS exposure and probable depressive symptoms (SHS in indoor public places: OR = 1.74, 95% CI: 1.32–2.29; SHS in homes: OR = 1.54, 95% CI: 1.20–1.97; SHS in indoor campuses: OR = 2.50, 95% CI: 1.76–3.56; SHS in outdoor campuses: OR = 2.17, 95% CI: 1.56–3.01). When differentiating these relations for different sources of campus SHS exposure, there were similar frequency–risk relationships for SHS exposure from smoking teachers (SHS in indoor campuses: OR = 2.18, 95% CI: 1.48–3.21; SHS in outdoor campuses: OR = 2.52, 95% CI: 1.69–3.77) and from smoking classmates (SHS in indoor campuses: OR = 3.36, 95% CI: 2.12–5.31; SHS in outdoor campuses: OR = 2.26, 95% CI: 1.56–3.29).

### 3.4. Relation between Binary SHS Exposure and Severe Depressive Symptoms

Compared with no exposure (Table 4), students with indoor SHS exposure experienced a higher prevalence of severe depressive symptoms (OR = 1.27; 95% CI 1.00–1.64, for SHS in homes; OR = 1.66; 95% CI 1.26–2.18, for SHS in indoor campuses). When examining the relations by source of exposure, similar positive relations were found for SHS exposure in indoor campuses from smoking teachers (OR = 1.52; 95% CI 1.13–2.06) and from smoking classmates (OR = 1.99; 95% CI 1.42–2.80). Notably, there was a significant relation between SHS exposure in outdoor campuses and severe depressive symptoms (OR = 1.55; 95% CI 1.20–2.01), and similar relations were observed for SHS exposure in outdoor campuses from smoking teachers (OR = 1.80; 95% CI 1.35–2.40) and from smoking classmates (OR = 1.51; 95% CI 1.13–2.03).

### 3.5. Relation between Frequency of SHS Exposure and Severe Depressive Symptoms

Table 5 indicates the frequency–risk relationships between SHS exposure and severe depressive symptoms. Ordinal frequency of indoor SHS exposure was significantly associated with severe depressive symptoms in a dose–response manner (SHS in indoor public places: *p* for linear trend = 0.036; SHS in homes: *p* for linear trend = 0.016; SHS in indoor campuses: *p* for linear trend <0.001; SHS in outdoor campuses: *p* for linear trend = 0.001). Additionally, there were increasing frequency–risk relationships between continuous frequency of SHS exposure and severe depressive symptoms (SHS in indoor public places: OR = 1.72, 95% CI: 1.17–2.55; SHS in homes: OR = 1.51, 95% CI: 1.06–2.16; SHS in indoor campuses: OR = 2.64, 95% CI: 1.67–4.16; SHS in outdoor campuses: OR = 2.21, 95% CI: 1.43–3.42). When differentiating these relations for different sources of campus SHS exposure, there were similar dose–response relationships for SHS exposure from smoking teachers (SHS in indoor campuses: OR = 2.29, 95% CI: 1.40–3.77; SHS in outdoor campuses: OR = 2.76, 95% CI: 1.67–4.54) and from smoking classmates (SHS in indoor campuses: OR = 3.04, 95% CI: 1.71–5.42; SHS in outdoor campuses: OR = 1.85, 95% CI: 1.12–3.07).

## 4. Discussion

This study builds on previous literature by addressing the potential frequency–risk relationships between SHS exposure and depressive symptoms among adolescents and differentiating these associations in setting-specific exposure and severity-specific outcomes. We found that there were monotonically increasing frequency–risk relationships between setting-specific (public places, homes, or campuses) SHS exposure and severity-specific (probable or severe) depressive symptoms. When examining these relations by source of exposure, there were similar positive and dose–response relationships for SHS exposure from smoking teachers and from smoking classmates.

Association between SHS exposure and depressive symptoms has been reported previously, but the findings are inconsistent [18,31,32,33]. The latest study of Chinese middle-aged women found that SHS exposure in homes was positively associated with depressive symptoms, but no association was observed for SHS exposure in indoor public places [32]. On the contrary, the study of Japanese workers indicated that SHS exposure at work was related with higher rates of depressive symptoms, but no relation was found for SHS exposure at home [18]. Additionally, the latest study on Chinese middle-aged women revealed a significant dose–response relationship between SHS exposure in homes and depressive symptoms [32], but the Korea National Health and Nutrition Examination Survey on men observed no dose–response association for home or workplace SHS exposure [34]. To date, only two studies have explored this relation in a population of adolescents, indicating that there was a significant relation between SHS exposure and depression or depressive symptoms [20,21]. However, it was unclear whether there is a dose–response relationship between SHS exposure and depressive symptoms among adolescents, and the potential relationships for setting-specific exposure and severity-specific outcomes were also unclear. Therefore, we aimed to assess the potential frequency–risk relationship among adolescents and differentiate this relationship in setting-specific (public places or homes) exposure and severity-specific (probable or severe) outcomes to make exposure and outcomes clearer. We found that there were frequency–risk relationships between setting-specific SHS exposure and severity-specific depressive symptoms. These findings point out the urgent need for comprehensive smoke-free legislation covering all public places and workplaces in Guangzhou to protect the public from SHS hazards, as called for in Article 8 of the Framework Convention on Tobacco Control. The above findings also suggest that the setting-specific associations between SHS exposure and depressive symptoms may differ in populations investigated, which may be due to differences in dose, frequency, and duration of SHS exposure in specific settings (such as homes, public places, and workplaces).

We note that much attention has been focused on SHS exposure in public places and in homes among adolescents, but there are still few studies regarding SHS exposure in indoor and outdoor campuses. Campus smoking bans were implemented in Guangzhou on 1 September 2010, and a full smoking ban (100% smoke-free) covers indoor and outdoor campuses. However, SHS exposure in indoor (22.7%) or outdoor (29.2%) campuses was still at a high level, which is consistent with results from other countries [35,36,37]. These findings suggest poor compliance with the full smoke-free ban in campuses. A few published studies have revealed that SHS exposure may be a risk factor of depressive symptoms, but only focused on SHS exposure in homes or in public places [18,21,32,33,34]. Of concern is that the potential relationship between SHS exposure in campuses and depressive symptoms is still unclear. Our study found positive relations and monotonically increasing dose–response relationships between setting-specific (indoor or outdoor campuses) SHS exposure and severity-specific (probable or severe) depressive symptoms. When differentiating these relationships for different sources of campus SHS exposure, there were similar dose–response relationships found for SHS exposure in campuses from smoking teachers or from smoking classmates. These findings provide more evidence for the potential influence of campus SHS exposure on depressive symptoms among adolescents, and support growing concern about SHS exposure in both indoor and outdoor campuses. These findings also indicate that the Guangzhou government needs to take measures to ensure the effective implementation of a full smoking ban in both indoor and outdoor campuses.

Although the nature of the relationship between SHS exposure and depression is uncertain, there are some possible mechanisms that may explain its relationship. Firstly, two biological mechanisms may explain the effects of SHS exposure on depression. One is the neurobiological mechanism observed in smokers and nonsmokers with SHS exposure. Several animal studies have revealed that nicotine exposure has acute and long-term effects on the dopamine system, which may lead to long-term imbalances in dopamine transport [9]. Lower levels of dopamine result from prolonged exposure to SHS, and have also been related to increased risk of negative mood or depression [10]. Another biological mechanism that relates SHS exposure to depressive symptoms is chronic inflammation [11,12]. Many studies have proposed that inflammatory cytokines induce the indoleamine 2,3-dioxygenase, which limits tryptophan and serotonin transport and may thus cause depression [13]. Secondly, there is increasing evidence suggesting that regular SHS exposure may be an indicator of a stressful working and living environment, and such chronic stress may be associated with the worsening of depressive symptoms through impaired neuroplasticity mechanisms and abnormal neurotrophic factors levels [8]. For example, chronic stress and major depression are associated with structural brain changes such as a loss of dendritic spines and synapses, reduced dendritic arborization, and diminished glial cells in the hippocampus [7].

Depression is one of the most common mental disorders and is considered a major public health problem. Moreover, adolescent depression is an antecedent of many adverse outcomes in adulthood, and globally imposes a significant economic burden not only on individuals with the condition, but also on their families and communities [38]. The present study found that 29.9% of the participants had probable depressive symptoms, which is similar to previous findings from other Asian countries (27.1% for university students in Turkey and 31.4% for Korean adolescents) [20,39]. However, the prevalence of depressive symptoms was low in adolescents in the United States (8.2% of females and 6.8% of males) [40]. These variations in the reported rates of depressive symptoms could be due, at least in part, to differences in populations investigated and in instruments for assessing depressive symptoms. Although there is no absolute consensus on which cutoff score is the best for discriminating clinically depressed persons from nondepressed persons, some authors have suggested that higher cutoff scores may reduce false-positive rates and improve agreement [41]. To make depressive symptoms more accurate, our study also used a higher cutoff point for depressive symptoms (that is, CES-D score ≥25 for severe depressive symptoms), on which has been placed particular emphasis in Asian countries and in some Hispanic ethnic groups [41,42,43]. Notably, the prevalence of severe depressive symptoms among adolescents was 9.4%, which was slightly higher than in Korean adults (8.7%) but lower than in Chinese college freshmen (17.6%) [26,44]. Note that screening is an important step in identifying depression in adolescents and securing appropriate treatment for adolescents experiencing severe depressive symptoms. There are a variety of depression screening tools available, including the Beck Depression Inventory-II, Patient Health Questionnaire-Adolescent Version, and Children’s Depression Inventory [45]. These tools take approximately 5 to 10 min to complete, including written assessments completed by the parent or adolescent and interview-style assessments administered by nurse practitioners.

There are some limitations that should be considered when interpreting the results. Firstly, the study design is cross-sectional, in which both exposure and outcomes are measured at the same time; therefore, we can only describe a potential association between SHS exposure and depressive symptoms, not a causal conclusion. Owing to the cross-sectional design of this study, it is possible that participants with depressive symptoms are more likely to report exposure than those without symptoms. Therefore, results from this study need to be confirmed in a longitudinal study. Secondly, smoking in previous studies was defined as “smoking 1 day or more within the past 30 days” or “smoking 1 cigarette or more within the past 7 days” [46,47]. However, smoking in this study was defined as “a history of smoking more than 100 cigarettes during the participant’s lifetime”, which is consistent with other previous studies but may underestimate the true prevalence of smokers [28,29,30]. Thirdly, SHS exposure in this study is based on self-reporting and has no biochemical measures (e.g., serum nicotine and cotinine). However, self-reported data obtained from population-based surveys was generally valid, apart from when there is a high demand for abstinence [48]. Additionally, self-reported SHS exposure allows the prevalence and frequency of exposure to be assessed, so we can explore the frequency–risk relationships and differentiate SHS exposure in different venues to explore setting-specific relations.

## 5. Conclusions

In conclusion, this study additionally contributes to the literature by exploring the frequency–risk relationships between SHS exposure and depressive symptoms in setting-specific exposure and severity-specific outcomes. We found that there were monotonically increasing frequency–risk relationships between setting-specific (public places, homes, or campuses) SHS exposure and severity-specific (probable or severe) depressive symptoms. These findings highlight the need for further longitudinal studies to establish the causal relationship and the need for smoke-free environments to protect the health of adolescents.

## Figures and Tables

**Table 1:** Demographic characteristics of the study participants.

Characteristics	*n*	%	Characteristics	*n*	%
Probable Depressive symptoms			Gender		
CES-D score < 16	2517	70.4	Female	1757	49.1
CES-D score ≥ 16	1058	29.6	Male	1818	50.9
Severe depressive symptoms			Monthly pocket money (¥)		
CES-D score < 25	3232	90.4	<100	2039	57.0
CES-D score ≥ 25	343	9.6	100–399	1125	31.5
SHS exposure in indoor public places			≥400	411	11.5
No	1806	50.5	Parents’ education		
Yes	1769	49.5	Neither from high school	1787	50.0
SHS exposure in homes			One from high school	622	17.4
No	2342	65.5	Both from high school	1166	32.6
Yes	1233	34.5	Prestigious schools		
SHS exposure in indoor campuses			No	1307	36.6
No	2763	77.3	Yes	2268	63.4
Yes	812	22.7	Grade		
SHS exposure in outdoor campuses			1–2	2329	65.2
No	2532	70.8	4–5	1246	34.8
Yes	1043	29.2	Only child		
Disease history			No	1353	37.8
0	885	27.6	Yes	2222	62.2
1	845	26.4	Negative life events		
2	627	19.6	No	2179	38.2
≥3	845	26.4	Yes	1345	61.8

*n*, number of participants; %, the proportion of participants surveyed; SHS, second-hand smoke; CES-D, the left for Epidemiologic Studies Depression.

**Table 2:** Relation between binary SHS exposure and probable depressive symptoms.

SHS Exposure	*n*	Probable Depressive Symptoms (%)	Unadjusted OR (95% CI)	Adjusted OR (95% CI) ^a^
SHS in indoor public places				
No	1806	428(23.7)	1.00	1.00
Yes	1769	630(35.6)	1.78(1.54–2.06)	1.38(1.16–1.63)
SHS in homes				
No	2342	619(26.4)	1.00	1.00
Yes	1233	439(35.6)	1.54(1.33–1.79)	1.24(1.04–1.47)
SHS in indoor campuses				
No	2763	761(27.5)	1.00	1.00
Yes	812	297(36.6)	1.52(1.29–1.79)	1.57(1.29–1.92)
SHS in indoor campuses from smoking teachers				
No	2940	837(28.5)	1.00	1.00
Yes	635	221(34.8)	1.34(1.12–1.61)	1.43(1.15–1.78)
SHS in indoor campuses from smoking classmates				
No	3149	873(27.7)	1.00	1.00
Yes	426	185(43.4)	2.00(1.63–2.46)	2.12(1.64–2.74)
SHS in outdoor campuses				
No	2532	674(26.6)	1.00	1.00
Yes	1043	384(36.8)	1.61(1.38–1.87)	1.49(1.24–1.79)
SHS in outdoor campuses from smoking teachers				
No	2917	816(28.0)	1.00	1.00
Yes	658	242(36.8)	1.50(1.25–1.79)	1.65(1.34–2.04)
SHS in outdoor campuses from smoking classmates				
No	2873	674(26.6)	1.00	1.00
Yes	702	384(36.8)	1.80(1.52–2.14)	1.55(1.26–1.91)

*n*, number of participants; SHS, second-hand smoke; OR, odds ratio; CI, confidence interval. ^a^ Adjusted for gender, grade, pocket money, parents’ education, negative life events, and disease history.

**Table 3:** Relation between frequency of SHS exposure and probable depressive symptoms.

Frequency of SHS Exposure	*n*	Probable Depressive Symptoms (%)	Unadjusted OR (95% CI)	Adjusted OR (95% CI) ^a^
SHS in indoor public places ^b^			2.62(2.07–3.31)	1.74(1.32–2.29)
SHS in indoor public places				
No exposure	1806	428(23.7)	1.00	1.00
1–4 days/week	1242	411(33.1)	1.59(1.36–1.87)	1.28(1.06–1.53)
5–7 days/week	527	219(41.6)	2.29(1.87–2.81)	1.66(1.30–2.10)
*p* for linear trend			<0.001	<0.001
SHS in homes ^b^			2.02(1.64–2.50)	1.54(1.20–1.97)
SHS in homes				
No exposure	2342	619(26.4)	1.00	1.00
1–4 days/week	570	176(30.9)	1.24(1.02–1.52)	0.98(0.78–1.24)
5–7 days/week	663	263(39.7)	1.83(1.53–2.19)	1.50(1.22–1.85)
*p* for linear trend			<0.001	<0.001
SHS in indoor campuses ^b^			2.21(1.66–2.94)	2.50(1.76–3.56)
SHS in indoor campuses				
No exposure	2763	761(27.5)	1.00	1.00
1–4 days/week	539	181(33.6)	1.33(1.09–1.62)	1.36(1.08–1.71)
5–7 days/week	273	116(42.5)	1.94(1.51–2.51)	2.13(1.56–2.91)
*p* for linear trend			<0.001	<0.001
SHS in indoor campuses from smoking teachers ^b^			1.94(1.42–2.66)	2.18(1.48–3.21)
SHS in indoor campuses from smoking teachers				
No exposure	2940	837(28.5)	1.00	1.00
1–4 days/week	412	130(31.6)	1.16(0.93–1.45)	1.26(0.97–1.63)
5–7 days/week	223	91(40.8)	1.73(1.31–2.29)	1.84(1.30–2.58)
*p* for linear trend			<0.001	<0.001
SHS in indoor campuses from smoking classmates ^b^			2.74(1.92–3.94)	3.36(2.12–5.31)
SHS in indoor campuses from smoking classmates				
No exposure	3149	873(27.7)	1.00	1.00
1–4 days/week	271	115(42.4)	1.92(1.49–2.48)	1.89(1.40–2.56)
5–7 days/week	155	70(45.2)	2.15(1.55–2.97)	2.67(1.77–4.04)
*p* for linear trend			<0.001	<0.001
SHS in outdoor campuses ^b^			2.20(1.69–2.87)	2.17(1.56–3.01)
SHS in outdoor campuses				
No exposure	2532	674(26.6)	1.00	1.00
1–4 days/week	704	248(35.2)	1.50(1.25–1.79)	1.37(1.11–1.68)
5–7 days/week	339	136(40.1)	1.85(1.46–2.33)	1.83(1.38–2.44)
*p* for linear trend			<0.001	<0.001
SHS in outdoor campuses from smoking teachers ^b^			2.21(1.60–3.06)	2.52(1.69–3.77)
SHS in outdoor campuses from smoking teachers				
No exposure ^c^	2917	816(28.0)	1.00	1.00
1–4 days/week	456	155(34.0)	1.33(1.07–1.64)	1.48(1.16–1.89)
5–7 days/week	202	87(43.1)	1.95(1.46–2.60)	2.16(1.50–3.10)
*p* for linear trend			<0.001	<0.001
SHS in outdoor campuses from smoking classmates ^b^			2.34(1.74–3.15)	2.26(1.56–3.29)
SHS in outdoor campuses from smoking classmates				
No exposure ^c^	2873	777(27.0)	1.00	1.00
1–4 days/week	451	181(40.1)	1.81(1.47–2.22)	1.42(1.11–1.81)
5–7 days/week	251	100(39.8)	1.79(1.37–2.33)	1.86(1.34–2.58)
*p* for linear trend			<0.001	<0.001

*n*, number of participants; SHS, second-hand smoke; OR, odds ratio; CI, confidence interval. ^a^ Adjusted for gender, grade, pocket money, parents’ education, negative life events, and disease history. ^b^ Logarithmic exposure (days/week) was used in the model.

**Table 4:** Relation between binary SHS exposure and severe depressive symptoms.

SHS Exposure	*n*	Severe Depressive Symptoms (%)	Unadjusted OR (95% CI)	Adjusted OR (95% CI) ^a^
SHS in indoor public places				
No	1806	137(7.6)	1.00	1.00
Yes	1769	206(11.6)	1.61(1.28–2.01)	1.25(0.98–1.61)
SHS in homes				
No	2342	195(8.3)	1.00	1.00
Yes	1233	148(12.0)	1.50(1.20–1.88)	1.27(1.00–1.64)
SHS in indoor campuses				
No	2763	234(8.5)	1.00	1.00
Yes	812	109(13.4)	1.68(1.32–2.13)	1.66(1.26–2.18)
SHS in indoor campuses from smoking teachers				
No	2940	262(8.9)	1.00	1.00
Yes	635	81(12.8)	1.49(1.15–1.95)	1.52(1.13–2.06)
SHS in indoor campuses from smoking classmates				
No	3149	274(8.7)	1.00	1.00
Yes	426	69(16.2)	2.03(1.52–2.70)	1.99(1.42–2.80)
SHS in outdoor campuses				
No ^b^	2532	204(8.1)	1.00	1.00
Yes	1043	139(13.3)	1.75(1.40–2.21)	1.55(1.20–2.01)
SHS in outdoor campuses from smoking teachers				
No ^b^	2917	250(8.6)	1.00	1.00
Yes	658	93(14.1)	1.76(1.36–2.27)	1.80(1.35–2.40)
SHS in outdoor campuses from smoking classmates				
No	2873	204(8.1)	1.00	1.00
Yes	702	139(13.3)	1.80(1.40–2.31)	1.51(1.13–2.03)

*n*, number of participants; SHS, second-hand smoke; OR, odds ratio; CI, confidence interval. ^a^ Adjusted for gender, grade, pocket money, parents’ education, negative life events, and disease history.

**Table 5:** Relation between frequency of SHS exposure and severe depressive symptoms.

Frequency of SHS Exposure	*n*	Severe Depressive Symptoms (%)	Unadjusted OR (95% CI)	Adjusted OR (95% CI) ^a^
SHS in indoor public places ^b^			2.52(1.79–3.55)	1.72(1.17–2.55)
SHS in indoor public places				
No exposure	1806	137(7.6)	1.00	1.00
1–4 days/week	1242	128(10.3)	1.40(1.09–1.80)	1.13(0.86–1.49)
5–7 days/week	527	78(14.8)	2.12(1.57–2.85)	1.44(1.02–2.02)
*p* for linear trend			<0.001	0.036
SHS in homes ^b^			1.87(1.36–2.56)	1.51(1.06–2.16)
SHS in homes				
No exposure	2342	195(8.3)	1.00	1.00
1–4 days/week	570	59(10.4)	1.27(0.94–1.73)	1.09(0.77–1.52)
5–7 days/week	663	89(13.4)	1.70(1.31–2.23)	1.45(1.07–1.95)
*p* for linear trend			<0.001	0.016
SHS in indoor campuses ^b^			2.61(1.76–3.86)	2.64(1.67–4.16)
SHS in indoor campuses				
No exposure	2763	234(8.5)	1.00	1.00
1–4 days/week	539	63(11.7)	1.43(1.07–1.92)	1.46(1.05–2.03)
5–7 days/week	273	46(16.9)	2.19(1.55–3.09)	2.10(1.40–3.12)
*p* for linear trend			<0.001	<0.001
SHS in indoor campuses from smoking teachers ^b^			2.39(1.55–3.69)	2.29(1.40–3.77)
SHS in indoor campuses from smoking teachers				
No exposure	2940	262(8.9)	1.00	1.00
1–4 days/week	412	42(10.2)	1.16(0.82–1.64)	1.27(0.87–1.85)
5–7 days/week	223	39(17.5)	2.17(1.50–3.13)	2.01(1.31–3.09)
*p* for linear trend			<0.001	0.001
SHS in indoor campuses from smoking classmates ^b^			2.83(1.76–4.56)	3.04(1.71–5.42)
SHS in indoor campuses from smoking classmates				
No exposure	3149	274(8.7)	1.00	1.00
1–4 days/week	271	43(15.9)	1.98(1.40–2.80)	1.89(1.27–2.82)
5–7 days/week	155	26(16.8)	2.11(1.36–3.28)	2.20(1.30–3.72)
*p* for linear trend			0.001	0.003
SHS in outdoor campuses ^b^			2.57(1.77–3.71)	2.21(1.43–3.42)
SHS in outdoor campuses				
No exposure	2532	204(8.1)	1.00	1.00
1–4 days/week	704	84(11.9)	1.55(1.18–2.02)	1.40(1.04–1.88)
5–7 days/week	339	55(16.2)	2.21(1.60–3.05)	1.94(1.32–2.83)
*p* for linear trend			<0.001	0.001
SHS in outdoor campuses from smoking teachers ^b^			2.87(1.86–4.41)	2.76(1.67–4.54)
SHS in outdoor campuses from smoking teachers				
No exposure	2917	250(8.6)	1.00	1.00
1–4 days/week	456	55(12.1)	1.46(1.07–2.00)	1.57(1.12–2.21)
5–7 days/week	202	38(18.8)	2.47(1.70–3.60)	2.36(1.52–3.66)
*p* for linear trend			<0.001	<0.001
SHS in outdoor campuses from smoking classmates ^b^			2.16(1.42–3.29)	1.85(1.12–3.07)
SHS in outdoor campuses from smoking classmates				
No exposure	2873	243(8.5)	1.00	1.00
1–4 days/week	451	63(14.0)	1.76(1.31–2.36)	1.43(1.02–1.99)
5–7 days/week	251	37(14.7)	1.87(1.29–2.72)	1.72(1.10–2.68)
*p* for linear trend			0.001	0.016

*n*, number of participants; SHS, second-hand smoke; OR, odds ratio; CI, confidence interval. ^a^ Adjusted for gender, grade, pocket money, parents’ education, negative life events, and disease history. ^b^ Logarithmic exposure (days/week) was used in the model.
